# Moon-Coal

**Published:** 1874-09

**Authors:** 


### MOON-COAL.

"A MAN in the moon," there may be, and possibly some green cheese; but there is no proof as yet that there is any coal there, which the inhabitants may shovel down to us, when ours is all burned up. But according to a late number of that most excellent paper, the *Scientific American*, a better thing has been lighted upon than a seam of coal a hundred miles thick, away out yonder in space; it is nothing more nor less than the discovery of doubling the yield of coal on this planet, thus : in getting coal out of the ground, and fitting it for market, about one-half is lost by its breaking up into small pieces and dust ; and at every coal mine, this waste has accumulated, amounting to many millions of tons ; a machine has been invented by which this waste, being put into one end of it, is turned out at the other end, two hundred and eighty feet distant, in the shape of little balls. This coal dust is so mixed with clay as to adhere closely, and is so covered with a kind of paint or glazing that in the handling of it the fingers are not soiled, no dust flies off, and the kindling is facilitated ; by being made into a spherical shape, spaces are given, when placed in the grate, for the circulation of air and a free draught, and, what is really a boon to man and blessing to the poor, can be furnished to consumers at one dollar a tun. Everyone of even moderate intelligence must feel gladdened at such an item of news as this ; and yet, the admirable paper mentioned, is constantly favoring its readers with the earliest information of similar practical matters, making it worth more than its subscription price for the moral and intellectual pleasure which it affords, to say nothing of the elevation it gives to the character. In the same number, one reads with a delight which is thrilling, the recital of events connected with the personal history of a man named James Lick, of whose very existence the great world outside of California was wholly ignorant. This gentleman has had an ambition for a life-time to facilitate telescopic discoveries, and having accumulated large wealth, he has been quietly and unostentatiously expending a portion of it in preparation for constructing a telescope, and placing it in a position which will result in making discoveries in the starry heavens, which will electrify the civilized world ; which, if anything mundane could, would call from their honored tombs all the Geelileo's, and Tycho Brach's, and Herschell's, and Rosse's and Mitchell's who have ever lived. A man may not be able to read a line with the naked eye, but he can do it easily with a pair of spectacles, because the object is enlarged, or brought nearer. An opera glass brings a beauty in a box most delightfully near to an old bachelor, almost near enough for a kiss ; and a telescope will bring a ship in sight, which before was almost invisible. Make a telescope long enough, and other things in proportion, and the moon may be brought almost near enough to see the people on it, if there be any there. To make such an one would cost, according to the estimate of some persons, a million of dollars. The great instrument lately sent to Washington will cost perhaps fifty thousand dollars. Mr. Lick has ordered the largest and most complete instrument that modern scientific knowledge can devise, with instructions to call on him to foot the bills. There is a grand man for you ! A common telescope is some two feet long ; now if one is constructed a hundred and twenty feet in length, to bring things to a focus, it will make a silk thread appear to be nearly thirty thousand times larger than it is ; it will increase the face of the moon to eight hundred millions of times its present extent, as we understand it ; in other words, we could see as much of the moon, could see it as clearly, as if we were within eight miles of it. The reader may inquire why not make the thing a little longer, so as to get near enough the moon to jump on to it, and make a picnic excursion ; it is sufficiently accurate to say, that the instrument could be easily made long enough, but according to present knowledge and skill, there is an insuperable difficulty in constructing a glass which will give a distinct image of an object at a greater distance. The importance and magnitude of the discoveries which might be made with such an instrument, properly located in a clear atmosphere, can scarcely be imagined ; it is a bliss to even contemplate the possibilities, and the name of James Lick will go down to the ages, carrying with it the admiration, the gratitude, and the respect of intelligent mankind to the end of time. But what a pity it is that a thousand families, where there is now but one, should not take such a paper as that named, at two or three dollars a year, and thus keep themselves informed of the progress of art and science ; of new developments, of new discoveries, and thus be in advance of the great mass of the people, and in this superior to them.

